# COVID-lateral Damage: Impact of the Post-COVID-19 Era on Procedural Training in Emergency Medicine Residency

**DOI:** 10.5811/westjem.59771

**Published:** 2023-09-01

**Authors:** Daniel Frank, Thomas Perera, Moshe Weizberg

**Affiliations:** * Zucker Hofstra School of Medicine, Northwell Health, South Shore University Hospital, Bay Shore, New York; † Zucker Hofstra School of Medicine, Northwell Health, North Shore/LIJ, Manhasset, New York; ‡ Zucker Hofstra School of Medicine, Northwell Health, Staten Island University Hospital, Staten Island, New York; § Maimonides Medical Center/Maimonides Midwood Community Hospital, Brooklyn, New York

## Abstract

**Introduction:**

Hospitalizations during the coronavirus 2019 (COVID-19) pandemic peaked in New York in March–April 2020. In the months following, emergency department (ED) volumes declined. Our objective in this study was to examine the effect of this decline on the procedural experience of emergency medicine (EM) residents compared to the pre-pandemic period.

**Methods:**

We conducted this multicenter, retrospective cohort study of patients seen and key procedures performed by EM residents at hospitals spanning three Accreditation Committee for Graduate Medical Education-approved EM residencies in New York City and Nassau County, NY. We obtained numbers of procedures performed during May–July 2020 and compared them to the same time period for 2019 and 2018. We a priori classified critical care procedures—cardioversion, central lines, chest tubes, procedural sedation, and endotracheal intubation. We also studied “fast-track” procedures—fracture/joint reduction, incision and drainage (I&D), laceration repairs, and splints.

**Results:**

Total number of critical care procedures in the months following the COVID-19 peak decreased from 694 to 606 (−12.7%, 95% confidence interval [CI] 10.3–15.4%), compared to an increase from 642 to 694 (+8.1%, 95% CI 6.1–10.5%) the previous year (difference −9.3%). Total number of fast-track procedures decreased from 5,253 to 3,369 (−35.9%, 95% CI 34.6–37.2%), compared to a decrease from 5,333 to 5,253 (−1.5%, 95% CI 1.2–1.9%) the year before (difference −36.3%). Specific critical care procedures performed in 2020 compared to the mean of 2019 and 2018 as follows: cardioversion −33.3%; central lines +19.0%; chest tubes −27.9%; procedural sedation −30.8%; endotracheal intubation −13.8%. Specific fast-track procedures: reductions +33.3%; I&D −48.6%; laceration repair −17.3%; and splint application −49.8%.

**Conclusion:**

Emergency medicine residents’ critical and fast-track procedural experience at five hospitals was reduced during the months following the COVID-19 peak in comparison to a similar period in the two years prior. Training programs may consider increasing simulation-lab and cadaver-lab experiences, as well as ED and critical care rotations for their residents to offset this trend.

Population Health Research Capsule What do we already know about this issue?

*The COVID-19 pandemic had dramatic effects on graduate medical education for procedure-based specialties such as surgery and interventional cardiology.*

What was the research question?

*What effect did the post-COVID-19 peak period have on the procedural experience in EM residency training?*

What was the major finding of the study?

*Index procedures decreased 33.2%, critical care procedures 12.7% (10.3–15.4%); and fast-track procedures 35.9% (34.6%–37.2%).*

How does this improve population health?

*Knowing the impact of COVID-19 on EM procedure training may spur programs to augment resident education impacted by interruptions.*

## INTRODUCTION

Graduate medical education (GME) of residents and fellows has been dramatically affected worldwide because of the coronavirus 2019 (COVID-19) pandemic. Similar influences on GME have been described during previous epidemics, natural disasters, and even in zones of war and conflict, although perhaps on a different scale.^1^^–^^6^ During the severe acute respiratory syndrome (SARS) epidemic of 2003, many educational activities were sacrificed, and outside clinical rotations were delayed due to fear of inter-site contamination when residents changed services.^1^^,^^5^ The ongoing conflict in Iraq has led to a significant impairment in quality of education, and the majority of trainees felt their safety to be at risk.^3^ The majority of trainees also reported their intent to leave Iraq after graduation, further crippling the critical healthcare safety net so many citizens rely on.^3^

When Hurricane Katrina struck Louisiana and Mississippi in 2005, hundreds of residents and fellows had their education disrupted. The Accreditation Council for Graduate Medical Education (ACGME) responded by quickly assisting in placing these trainees, either temporarily or permanently, in other programs that could meet their educational needs. The resulting policies provided the framework for institutions to respond to the needs of their GME programs during Hurricanes Ike in 2008 and Harvey in 2017.^4^^,^^6^

Early literature on effects on GME of the COVID-19 pandemic focused on procedure-based specialties, such as surgery, urology, interventional radiology, interventional cardiology, ophthalmology, and urology.^7^^–^^11^ Cancellation of elective procedures and redeployment of residents and fellows created a dramatic reduction in cases needed for adequate training. Surgical programs especially reported decreased clinical experience, reduced case volume, and disrupted education activities as major concerns.^12^ Additionally, the ACGME allowed institutions to apply for “emergency status.” This eased some of the mandatory training requirements that were normally in place. To mitigate this problem, the American Board of Surgery announced they would accept a 10% reduction in operative cases from graduating residents, while other specialties suggested training might have to be extended to ensure adequate training, especially in a prolonged epidemic scenario.^10^^,^^13^^,^^14^

Emergency medicine (EM) trainees were similarly affected by the overall decrease in patient volumes that many emergency departments (ED) experienced throughout the pandemic. Decreased case variety, fewer procedures, and fewer patients with serious diagnoses all negatively impacted resident training during the early pandemic period.^15^^,^^16^

Throughout the world, GME programs quickly responded with innovation and utilization of online resources during the pandemic to minimize disruptions in resident education. Most didactics, journal clubs, and case reviews moved online, and large conferences became virtual. Many outpatient encounters were taking place via telemedicine platforms. Some surgical programs even started working with simulators built in house, while others developed virtual reality technology in an effort to maintain technical skills.^8^^,^^9^^,^^11^^,^^14^^,^^17^^,^^18^

Emergency medicine training programs similarly confronted challenges in resident education since the onset of the pandemic. All resident training depends in large part on patient encounters to gain experience and develop clinical acumen and technical skill. Traditionally, postgraduate year (PGY)-1 EM residents would average 0.7 patient encounters per hour, while PGY-3 residents could see 1.3 patients per hour.^19^ The pandemic, however, caused a dramatic reduction in ED patient volumes, especially among pediatric patients.^20^ During the early pandemic period, ED visits in the United States were 42% lower than the same period a year earlier, with the largest proportional decline in patients 14 years or younger.^21^ As a result of the pandemic, EM residents’ critical procedural experiences also decreased.^22^ Moreover there was a significant decline in all non-COVID-19 related patient presentations, with significantly less resident exposure to cardiac, psychiatric, surgical, and neurosurgical cases.^23^

Hospitalizations for COVID-19 peaked in New York City in March and April 2020. In the months following, ED volumes declined. Our objective was to study the effect of this decline on the procedural experience of EM residents compared to the pre-pandemic period. Through a multicenter study, we sought to describe the volume, types, and acuity of cases seen by EM residents during the months following the COVID-19 peak within three separate EM training programs in New York City and Nassau County. Such information can inform decisions on how best to augment resident education and maintain quality of training during program interruptions and times of reduced patient volume, clinical exposure, and procedural experience.

## METHODS

### Study Design

This was a multicenter, retrospective cohort study of the number of patients seen and number of procedures performed by EM residents during the months following the COVID-19 peak. We compared these numbers to the same period in the two previous years. The study was reviewed by the institutional review board and was deemed to be not human subjects research.

### Study Setting and Population

This was a multicenter study at five hospitals spanning two ACGME-approved EM residencies and one combined EM-IM residency in New York City and Nassau County. The hospitals include three tertiary care centers, one community hospital, and one children’s hospital. Prior to COVID-19, the four adult EDs ranged in patient volume from 33,000 to 102,000 patients per year, and the pediatric ED had a volume of 60,000 patients per year. The residency programs include two EM programs in the PGY 1–3 format, as well as one combined EM-internal medicine (IM) program. The number of residents at each program are 30 and 68 for the EM programs and 10 for the EM-IM program.

Our study took place from May–July 2020. These dates were chosen because they represented the initial period following the major first wave of COVID-19 patients in New York. As a comparison group, we collected data from the same months in the preceding two years.

### Study Protocol

We performed an electronic data query to extract data from our health system’s electronic health record (EHR). We used standard record review practices.^24^ Variables obtained from the electronic data query included patient demographics, disposition, authorship of physician note, diagnosis, and chief complaint. Deidentified data were stored in an Excel spreadsheet (Microsoft Corp, Redmond, WA) on a secure server. We reviewed the total number of patients seen by EM residents during the study period, as well as the admission percentage. We identified patients seen by residents based on the authorship of the physician note. This was to ensure each record would only be counted once. We omitted patients seen by physician assistants, nurse practitioners, or by attendings primarily.

We reviewed our EHR billing data for the number of predetermined procedures performed by EM residents during this period. Billing data was captured from procedure notes written by the physicians. We studied critical care procedures, specifically electrical cardioversion, central lines, chest tubes, procedural sedation, and endotracheal intubation. These are key procedures required by the Residency Review Committee in Emergency Medicine (RRC-EM). We also studied “fast-track” procedures, including fracture/joint reduction, incision and drainage (I&D), laceration repairs, and splints. We compared the numbers of procedure performed to the same period in the previous two years.

### Measurements or Key Outcome Measures

Numbers of patients seen and procedures performed in the months following the COVID-19 peak were compared to the numbers during the same period for 2019 and 2018. The primary outcome was the change in the total number of index procedures during the months following the COVID-19 peak as compared to the mean of the two previous years. Secondary outcomes included total number of patients seen, and changes in the number of critical care and fast-track procedures.

### Data Analysis

We calculated statistics using SAS statistical software (SAS Institute Inc, Cary NC). Percentage of admissions was compared using chi-square tests.

## RESULTS

Total number of patients seen by residents during the months following the COVID-19 peak was 33,246, compared to 49,316 in 2019 (−32.6%) and 49,748 in 2018 (−0.9%) (Table 1). Admission percentage was 32.0% in the months following the COVID-19 peak compared to 28.5% in 2019 and 28.5% in 2018.

**Table 1.** Comparison of patient volume for the COVID-19 study period and the control period.

	2020	2019	2018	*P*-value
Total # of patients seen by residents	33,246	49,316	49,748	<0.001
Admission percentage	32.0%	28.5%	28.5%	<0.001

The total number of index procedures decreased from 5,947 in 2019 to 3,975 in 2020 (−33.2%); compared to virtually no change the previous year (5,975 to 5,947). Total critical care procedures in the months following the COVID-19 peak decreased from 694 in 2019 to 606 in 2020 (−12.7%, 95% confidence interval [CI] 10.3–15.4%), compared to an increase from 642 to 694 (+8.1%, 95% CI 6.1–10.5%) the previous year (difference −9.3%). Total fast-track procedures decreased from 5,253 in 2019 to 3,369 in 2020 (−35.9%, 95% CI 34.6–37.2%), compared to a decrease from 5,333 to 5,253 (−1.5%, 95% CI 1.2–1.9%) the year before (difference −36.3%) (Table 2).

**Table 2.** Comparison of total procedures for the study period and the control period.

	2020	2019	2018	Difference 2020 from mean of 2018/2019
Total critical care procedures	606	694	642	−9.3%
Total fast-track procedures	3,369	5,253	5,333	−36.3%
Total procedures	3,975	5,947	5,975	−33.3%

The data for the individual critical care and fast-track procedures are presented in Table 3 and Table 4. During the study period, there was a notable decrease in the critical care procedures of cardioversion, chest tube, procedural sedation, and endotracheal intubation. The only critical care procedure that demonstrated an increase was central line placement. There was also a decrease noted in fast-track procedures of I&D, laceration repair, and splint application. The only fast-track procedure that demonstrated an increase was orthopedic reductions.

**Table 3.** Comparison of critical care procedures for the study period and the control period.

Procedure	2020	2019	2018	Difference 2020 from mean of 2018/2019
Cardioversion	7	11	10	−33.3%
Central line	244	212	198	+19.0%
Chest tube	22	27	34	−27.9%
Procedural sedation	126	190	174	−30.8%
Endotracheal intubation	207	254	226	−13.8%

**Table 4.** Comparison of fast-track procedures for the study period and the control period.

Procedure	2020	2019	2018	Difference 2020 from mean of 2018/2019
Reductions	36	32	22	+33.3%
Incision and drainage	284	551	553	−48.6%
Laceration repair	1,739	2,090	2,115	−17.3%
Splint application	1,310	2,580	2,643	−49.8%

## DISCUSSION

March–April 2020 represented the peak of COVID-19 cases in the first wave in the New York metropolitan area. During this time EDs were flooded with extremely sick patients. Residents in EM were often managing multiple critical patients simultaneously. They were also exposed to large-scale death, likely for the first time in their careers. Residents were performing large numbers of intubations and medical resuscitations. This had a dramatic impact on their training.

The immediate post-COVID-19 era, however, saw a dramatic decline in ED volume. This was likely multifactorial, with patients afraid to come to EDs out of fear of contracting COVID-19, as well as their adherence to stay-at-home orders. This also had an effect on EM residency training. Our study found that numbers of ED patients seen by residents decreased by 32.6%. At the same time, admission percentage increased by 3.5%, suggesting that lower acuity patients were not coming to the ED. The numbers of both critical care procedures and fast-track procedures decreased during this period as compared to the previous year. The most dramatic changes were noted in fast-track procedures, with notable decreases in I&D, laceration repair, and splint application.

These findings can have a significant impact on resident education. If this trend continues, we may see residents graduating from training with significantly less procedural experience, especially in the realm of fast-track procedures. Our study can inform decisions on how best to augment resident education and maintain quality of training during program interruptions and times of reduced patient volume, clinical exposure, and procedural experience.

These results are likely multifactorial. In the immediate post-COVID-19 era, many people were afraid to come to the hospital due to a fear of contracting the disease. Alternative locations for care became available including telemedicine and urgent care centers. Finally, patients may have chosen not to seek care at all.

Residency programs are encouraged to look at the numbers of procedures that their residents are performing, especially fast-track type procedures. As these procedure numbers are not required to be reported to the ACGME, there is a possibility that residency programs will not be aware of the individual numbers in their own programs if not specifically examined. Programs that find their residents have a deficiency in the number of fast-track procedures may wish to supplement their experience with procedural training in the simulation lab or cadaver lab.

## LIMITATIONS

The study has several limitations, First, it took place in one region of the country, namely New York. These findings may not be generalizable to the rest of the US. However, we did study three different programs at five different hospitals spanning four different counties in the area. Second, this was a retrospective cohort study and, therefore, our data is somewhat limited. It is possible that if the data would have been collected prospectively, we would have had a more complete and more accurate dataset.

Our results were obtained from billing data, which is captured from procedure notes written by the physicians. It is possible that some procedures were performed without a procedure note being written. In that scenario, the procedure would not have been captured in our dataset. However, our billers carefully review all charts and encourage physicians to complete all procedure notes if they have not been completed. In addition, the same methods for collecting procedure data were used in both the study population and the control population.

Additionally, we only looked at data for three months. It is possible that what we observed was a temporary phenomenon and as volumes in EDs began to return, procedural availability has improved as well. Finally, we did not look at procedure numbers during the actual COVID-19 peak. It is possible that the increase in critical care procedures during this period offset the decrease we saw in the study period. However, this would not account for the decrease in fast-track procedures, which essentially went away during the COVID-19 peak.

## CONCLUSION

The post-COVID-19 era resulted in lower patient volume and less availability of certain procedures for EM residents in New York City and Nassau County. This phenomenon should be watched closely to determine whether trends appear. If so, interventions may need to be instituted to supplement resident training.
